# Acute Kidney Injury in Hospitalized Cancer Patients: Single-Centre Real-Life Analysis of Incidence and Clinical Impact

**DOI:** 10.3390/jcm15020690

**Published:** 2026-01-15

**Authors:** Pasquale Esposito, Francesca Cappadona, Annarita Bottini, Elisa Russo, Giacomo Garibotto, Vincenzo Cantaluppi, Francesca Viazzi

**Affiliations:** 1 Unit of Nephrology, Dialysis and Transplantation, IRCCS Azienda Ospedaliera Metropolitana (IRCCS AOM) San Martino, 16132 Genoa, Italy; francesca.cappadona@hsanmartino.it (F.C.); elisa.russo@unige.it (E.R.); francesca.viazzi@unige.it (F.V.); 2 Department of Internal Medicine and Medical Specialties (DIMI), University of Genova, Viale Benedetto XV, 16132 Genova, Italy; annaritabottini@hotmail.it; 3 Nephrology and Kidney Transplantation Unit, Department of Translational Medicine (DIMET), University of Piemonte Orientale (UPO), AOU Maggiore Della Carità, 28100 Novara, Italy; vincenzo.cantaluppi@med.uniupo.it

**Keywords:** acute kidney injury, hematologic cancers, hospitalization, neoplasms, onconephrology, sepsis

## Abstract

**Background:** Acute kidney injury (AKI) is a frequent and clinically relevant complication in cancer patients, with highly variable incidence. AKI increases morbidity and mortality, prolongs hospitalization, and may limit access to oncologic therapies. This study evaluated the incidence, risk factors, and outcomes of AKI in hospitalized cancer patients. **Methods:** We retrospectively analyzed patients admitted between 1 January 2016 and 31 December 2019. Individuals with cancer were identified and categorized into three groups: hematologic malignancies, solid cancers with metastases, and solid cancers without metastases. Demographic, clinical, and laboratory data were collected, and AKI was defined and staged according to KDIGO criteria, evaluating serum creatinine changes. **Results:** Among 56,390 hospitalized patients, 6723 (11.9%) had a cancer diagnosis. AKI incidence was significantly higher in cancer versus non-cancer patients (30.1% vs. 19.6%). Hematologic cancers showed the highest incidence (39.3%). Among hematologic patients, ICU admission, sepsis, and diabetes were strongly associated with AKI. In non-metastatic solid cancers, more conventional factors—including female sex, older age, sepsis, and ICU admission—were significant predictors. In contrast, in metastatic solid cancers, traditional AKI risk factors did not correlate with increased AKI occurrence. In cancer patients overall, AKI per se did not increase mortality risk; however, stage 3 AKI was associated with significantly higher mortality (HR 1.37, 95% CI 1.13–1.66, *p* < 0.001). **Conclusions:** AKI is common in hospitalized cancer patients, with specific patterns and heterogeneous risk factors and impact on outcomes. Implementation of tailored preventive strategies and early recognition are necessary to mitigate progression and improve clinical trajectories.

## 1. Introduction

Over the past decade, cancer outcomes have improved significantly thanks to the development of novel anticancer agents and advances in supportive care. Despite this progress, acute kidney injury (AKI) remains one of the most common and clinically relevant conditions observed in patients with cancer, with the potential not only to worsen oncologic outcomes but also to restrict eligibility for systemic therapies, extend hospital stays, and increase healthcare resource utilization [1]. Known risk factors include advanced cancer stage, female sex, intravascular volume depletion, pre-existing chronic kidney disease, and diabetes mellitus. Severity of illness and baseline functional status also significantly influence the risk of AKI and its prognosis [2]. In addition to causes observed in the general population, AKI in cancer patients is often driven by malignancy-specific and treatment-related mechanisms [3]. These include drug-induced renal injury, tumor lysis syndrome, direct tumor infiltration of the kidneys, and paraneoplastic syndromes [4,5]. Reported AKI incidence among oncology patients varies widely, ranging from 12% to over 50%, with consistently higher rates in hematologic malignancies than in solid tumors, and the highest frequencies seen in critically ill individuals [6,7]. In a large Danish cohort of 37,267 incident cancer cases, the 1-year AKI risk was 17.5%, rising to 27% at 5 years, with the highest incidences observed in kidney cancer, liver cancer, and multiple myeloma [8]. Observational data indicate that AKI occurs in up to 60% of adults with hematologic cancers, approximately 45% of those with kidney cancer, and around 30% of those with liver cancer [9]. In pediatric populations, the highest incidences are reported in urinary tract tumors, liver cancers, and retroperitoneal malignancies [10]. To date, AKI prevention and management rely largely on clinical judgment. Although international guidelines provide a series of rational recommendations for the early identification and treatment of AKI, many are opinion-based, and studies evaluating their real-world effectiveness have yielded inconsistent or negative results [11,12]. One major reason for the limited success of generalized approaches is that AKI is not a single disease entity but rather a syndrome encompassing multiple pathogenic mechanisms across diverse patients and clinical settings [13]. Detailed characterization of AKI phenotypes may therefore represent a key strategy to overcome the limitations of uniform preventive and therapeutic approaches [14]. This need for a more granular understanding is particularly relevant for cancer-associated AKI, as oncologic patients display specific clinical profiles, unique pathogenic contributors, and potentially distinct outcomes compared with the general AKI population. Based on these considerations, in the present study, we analyzed AKI epidemiology and outcomes in a large cohort of hospitalized patients with cancer, further stratifying them according to the underlying type of malignancy.

## 2. Materials and Methods

### 2.1. Study Design and Patients’ Enrolment

This study is part of a larger initiative aimed at evaluating general AKI epidemiology in Italian hospitals, supported by the AKI & CRRT Project Group of the Italian Society of Nephrology (SIN) [15]. Within the overall multicenter dataset, we focused on the data on cancer patients collected at our institution only. We therefore conducted a retrospective observational study including adult patients hospitalized at IRCCS Policlinico “San Martino”, Genoa (Italy), between 1 January 2016 and 31 December 2019. Only the first hospitalization during the study period was considered for each patient.

Diagnoses and comorbidities were retrieved from administrative data reported in the Hospital Discharge Form (HDF), coded according to the International Classification of Diseases, 9th Revision, Clinical Modification (ICD-9-CM). Patients with chronic kidney disease (CKD) stage 4 or 5 were excluded. From the overall hospitalized population, we identified and analyzed patients with cancer based on ICD-9-CM diagnosis codes recorded in the HDF. Analyses were performed in the overall oncology cohort and in subgroups defined by malignancy type. Specifically, patients were stratified into three categories: (I) hematological malignancies (e.g., lymphoma, leukemia, multiple myeloma); (II) solid tumors with metastases; and (III) solid tumors without metastases. The study protocol was approved by the local Institutional Review Board (Comitato Etico Regionale Liguria, approval no. 515/2020; 3 March 2021), which waived the requirement for informed consent. The study was conducted in accordance with the Declaration of Helsinki.

English editing support was provided with the assistance of a large language model (LLM), ChatGPT (OpenAI, GPT-5.2). The authors are responsible for the final content.

### 2.2. Data Collection and Definition

From the hospital electronic database, we extracted demographic, clinical, and laboratory data, including cancer type (solid vs. hematologic), metastatic status (with vs. without), and outcomes.

AKI occurrence and staging were assessed using changes in serum creatinine (sCr) during hospitalization. For each patient, sCr values at admission and discharge, as well as the peak and nadir sCr during the hospital stay, were retrieved. Patients were included in the analysis only if at least two sCr measurements were available. To standardize AKI ascertainment in this retrospective dataset, the lowest sCr value recorded during hospitalization was used as the baseline. AKI presence and stage were determined by the ratio between peak and baseline sCr (peak sCr/lowest sCr). AKI was defined according to the “extended” KDIGO Clinical Practice Guideline, based solely on sCr changes and without applying specific time windows between measurements [16]. AKI severity was categorized according to KDIGO stages: stage 1 (1.5–1.9 times baseline sCr), stage 2 (2.0–2.9 times baseline sCr), and stage 3 (≥3.0 times baseline sCr or need for dialysis). Urine output criteria were not applied due to the retrospective design and limited availability of these data.

### 2.3. Outcomes

The following outcomes were considered for the overall cohort of cancer patients and different cancer groups: (a) incidence and risk factors of in-hospital AKI development, (b) clinical outcomes in terms of mortality rate, length of hospital stay (LOS), ICU admission, and sCr levels at hospital discharge.

### 2.4. Statistical Analysis

Normally distributed variables are presented as mean ± SD and were compared using an independent or paired *t*-test when appropriate. Comparisons between groups were made by analysis of variance. Non-normally distributed variables are presented as median plus interquartile range and were compared using non-parametric tests. Comparisons of proportions were made using the χ^2^-test or Fisher’s exact test when appropriate. Logistic regression analysis was used to describe the relationship between clinical variables of clinical relevance and the occurrence of AKI, as well as the other clinically relevant outcomes (e.g., ICU admission). The results of logistic regression analysis are reported as Odds Ratios (ORs) with 95% confidence intervals (95% CI). Univariate and multivariate Cox regression analyses were used to determine the effect of the variables of interest on in-hospital mortality. Time-to-event was calculated as the time elapsed from hospital admission to the time of death or hospital discharge, with right-censoring at 90 days.

Results of time-to-event analysis are reported as Hazard Ratios (HRs) along with their 95% confidence intervals (95% CI). Covariates included all available clinical variables with biological plausibility. A power analysis indicated that the database sample size (n = 56,390) was sufficient to avoid a Type II error (beta error), even after stratification by AKI status. Statistical calculations were performed by the STATA package, version 14.2 (StataCorp, 4905 Lakeway Drive, College Station, TX, USA). The null hypothesis was rejected for *p*-values < 0.05.

## 3. Results

### 3.1. Patients’ Characteristics

We collected data from 56,390 hospitalized adults, with a mean age of 70.1 ± 18.7 years; 52.2% were female. Among them, 6723 patients (11.9%) had an oncological diagnosis. Cancer patients were older than the non-oncologic population (73.0 ± 12.4 vs. 69.7 ± 19.4 years) and included a lower proportion of females (43.7% vs. 53.3%). At hospital admission, patients with cancer had higher sCr levels than non-oncologic individuals (1.21 ± 1.05 mg/dL vs. 1.13 ± 0.86 mg/dL, *p* < 0.001). In the oncology cohort, 8.9% had diabetes mellitus (DM), 5.5% had heart failure (HF), and 6.3% had pre-existing CKD. While diabetes prevalence was similar between groups, HF and CKD were both significantly more common in non-oncologic patients (9.7% and 8.2%, respectively). Most patients were admitted to low- or intermediate-intensity care units. ICU admission occurred in 1.33% of cancer patients and 5.53% of non-cancer patients. Sepsis during hospitalization was slightly more frequent among non-oncologic patients (5.6% vs. 4.7%).

Table 1 summarizes the principal characteristics of the two populations.

The oncologic cohort was stratified into three categories: hematologic malignancies (16.5%, n = 1107), solid cancers without metastases (70.9%, n = 4767), and metastatic solid cancers (12.6%, n = 849). Patients with hematologic malignancies were older than those in the other groups (73.9 ± 11.8 years).

DM prevalence did not differ significantly across cancer types. HF was more frequent in non-metastatic solid cancer, while CKD prevalence was highest among hematologic patients (9.6% vs. 6.1% and 2.7%, *p* < 0.001), who also showed the highest sCr levels at admission (1.39 ± 1.21 mg/dL). Sepsis occurred far more commonly in hematologic cancers (19.5%) compared with solid cancers with or without metastases. Detailed characteristics are reported in Table 2.

### 3.2. AKI Incidence and Staging

**Figure 1:**
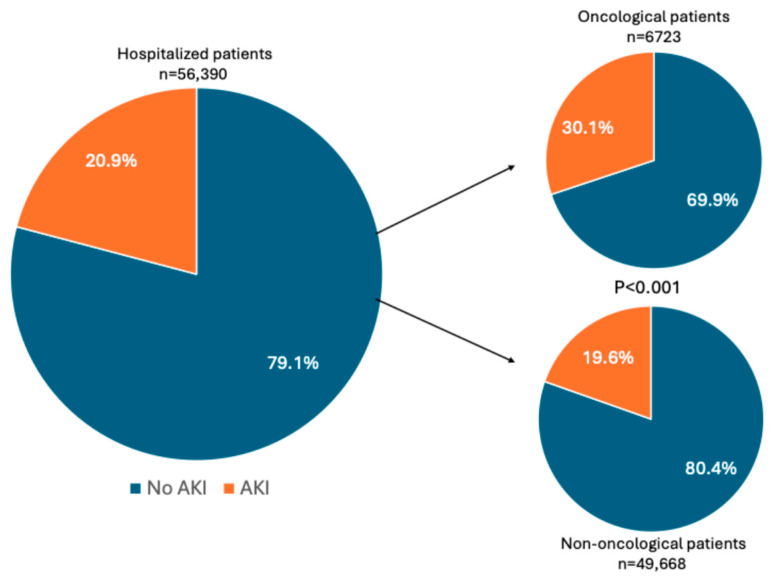
AKI incidence in the overall population and oncological/non-oncological patients.

AKI developed in 11,771 patients (20.9%) (Figure 1). Oncologic patients had a higher AKI incidence than non-oncologic subjects (30.1% vs. 19.6%, *p* < 0.001). Although the distribution of AKI stages was similar between groups, stage 3 AKI occurred more frequently in the cancer population (16.4% vs. 14.6%, *p* = 0.04) (Table 3).

**Figure 2:**
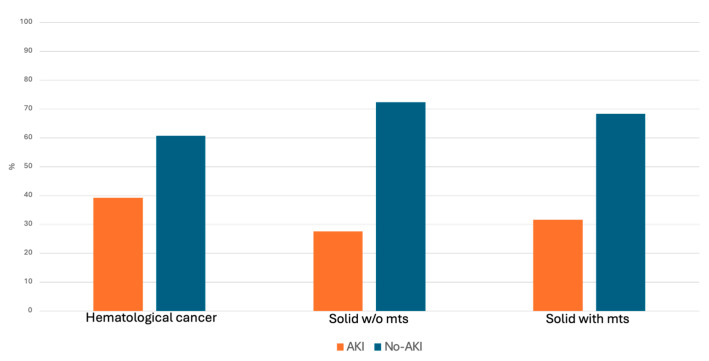
AKI incidence in oncological subgroups. Abbreviations: mts = metastases, AKI = Acute Kidney Injury.

Subgroup analysis showed substantial variation in AKI incidence among different cancer types. Hematologic cancer experienced the highest frequency (39.3%), followed by metastatic solid cancer (31.7%) and non-metastatic solid cancer (27.6%) (Figure 2). Stage distribution was similar across subgroups, although metastatic solid cancers demonstrated a lower proportion of stage 3 AKI (Table 4).

### 3.3. AKI Risk Factors in Oncological and Non-Oncological Populations

Multivariate logistic regression showed that, in patients with cancer, the strongest predictors of AKI were sepsis (OR 4.96, 95% CI 3.3–7.4; *p* < 0.001), ICU admission (OR 3.89, 95% CI 2.7–5.6; *p* < 0.001), and female sex. Instead, in non-oncological patients, beyond these factors, AKI risk was also associated with age and HF. Finally, in both groups, CKD appeared inversely associated with AKI, and DM was not a significant predictor (Table 5).

### 3.4. AKI Risk Factors in Oncological Subgroups

Analysing more in detail the predictor factors for AKI development in the subgroups of oncological patients, we found that in haematological patients, sepsis, diabetes, and ICU admission were significantly associated with AKI risk. A similar pattern was observed in solid non-metastatic cancers. Instead, in the metastatic subgroup, none of the variables reached statistical significance; however, both sepsis and ICU admission showed associations with increased risk of AKI (e.g., sepsis OR 4.28). These associations did not reach statistical significance because of wide confidence intervals, indicating limited precision of the estimates, likely due to the small number of metastatic patients with sepsis and/or ICU admission.

**Figure 3:**
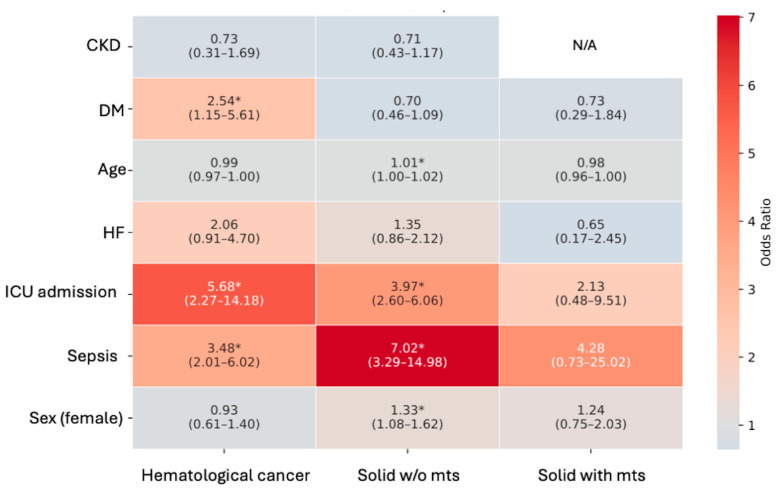
Heatmap displaying odds ratios (ORs) and 95% confidence intervals (CIs) for risk factors associated with acute kidney injury (AKI) across oncological subgroups. Colours represent the magnitude and direction of the association (blue for OR < 1 and red for OR > 1). * *p* < 0.05. N/A: In patients with solid metastatic cancer, CKD was omitted from the multivariable model due to perfect prediction. Abbreviations: AKI = acute kidney injury, mts metastases, CKD = chronic kidney disease; HF = heart failure; DM = diabetes mellitus; ICU = intensive care unit.

Finally, across all subgroups, pre-existing CKD was not a statistically significant predictor of AKI (Figure 3).

### 3.5. Outcomes and Associations with AKI Development

The in-hospital mortality rate was significantly higher in the oncological population than in those without cancer (16.52% vs. 7.21%, *p* < 0.001) (Table 6). Median LOS observed was 9 (4–15) days; it was significantly longer in oncologic patients [12 (7–21) days vs. 8 (4–15), *p* < 0.001]. In oncologic patients, there was a higher prevalence of LOS ≥ 15 days (42.3% vs. 25.2%, *p* < 0.001). Finally, sCr at discharge was significantly higher in the oncological group.

**Figure 4:**
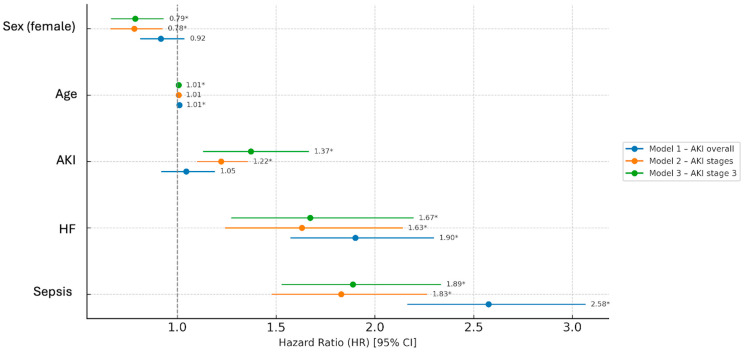
Forest plot based on multivariate Cox regression analysis in the whole oncological patient cohort, divided by different AKI models. Abbreviations: HR = Hazard Ratio, CI = Confidence Interval, AKI = Acute Kidney Injury, HF = Heart Failure, * *p* < 0.05.

Cox analysis to evaluate the factors associated with the risk of in-hospital mortality was performed using three AKI models: model 1 considered overall AKI stages development, model 2 evaluated AKI stages, and model 3 evaluated only the occurrence of stage 3 AKI. In the univariate model, in non-oncological patients, AKI increased the mortality risk approximately twofold (HR 2.11, 95% CI 1.97–2.27, *p* < 0.001). This finding was also confirmed in the multivariate analysis, which additionally showed that in this group, the mortality risk was significantly affected by each considered variable (with a protective role of female sex), while developing sepsis was the most important mortality risk factor. In contrast, AKI had a weaker effect in oncologic patients (HR 1.18, *p* = 0.007). After adjustment, AKI did not independently predict mortality in cancer patients; however, AKI severity, expressed both as AKI stage and AKI stage 3, was associated with increased mortality risk. However, also in this cohort, sepsis was consistently the strongest predictor across all models (Table 7, Figure 4).

### 3.6. Outcomes in Oncological Patients’ Subgroups

In the univariate model, in non-oncological patients, AKI increased the mortality risk approximately twofold (HR 2.11, 95% CI 1.97–2.27, *p* < 0.001). This finding was also confirmed in the multivariate analysis, which additionally showed that in this group, the mortality risk was significantly affected by each considered variable (with a protective role of female sex), while developing sepsis was the most important mortality risk factor. In contrast, the in-hospital mortality rate was higher in metastatic cancer, compared to haematologic patients and solid cancer without metastases, whose mortality rate was the lowest (22.9% vs. 18.9% vs. 14.8%, respectively, *p* < 0.001) (Table 8).

Patients with haematological neoplasm had a longer hospitalization, 14 days (8–24), compared to those with solid cancer, 12 days (7–20) (*p* = 0.0001). There were no differences in LOS in patients with solid neoplasia between the groups with or without metastasis.

LOS ≥ 15 days was most frequent in hematologic patients (48.7%), while sCr at discharge was also highest in this subgroup. In the haematological subgroup, univariate analysis showed that AKI development did not increase mortality risk (HR 1.19, 95% CI 0.89–1.60, *p* = 0.23), nor did AKI severity impact (HR 1.19, 95% CI 0.96–1.49, *p* = 0.11). Only stage 3 AKI increased mortality risk in this population (HR 1.55, 95% CI 1.06–2.28, *p* = 0.02). Multivariate analysis confirmed that stage 3 AKI was independently associated with higher mortality risk (HR 1.78, 95% CI 1.21–2.61, *p* = 0.03) (Table 9).

**Figure 5:**
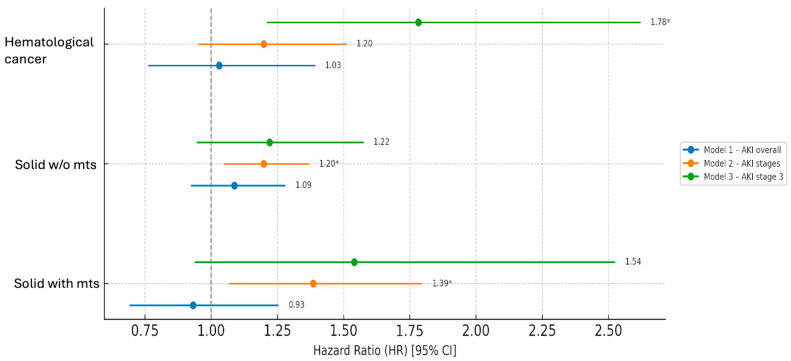
Forest plot based on Cox regression analysis in oncological subgroups, divided by AKI models. Adjusted for sex, age, HF, and sepsis. Abbreviations: mts = metastases, * *p* < 0.05.

In the group of patients with non-metastatic solid cancer, the univariate model showed that developing AKI (HR 1.25, 95% CI 1.07–1.46, *p* < 0.01), AKI severity, and stage 3 AKI correlated with mortality risk. In multivariate analysis, AKI severity still remained significantly associated (HR 1.2, 95% CI 1.05–1.37, *p* = 0.007). In this group, sepsis was the most important risk factor for mortality in all analysis models. Finally, in cancer patients with metastases, at multivariate analysis, only AKI severity, but not the presence of AKI per se, was associated with mortality risk, while other factors did not impact mortality, except for sepsis in model 1 (Figure 5).

## 4. Discussion

In this large cohort of hospitalized adults, we observed that AKI is a frequent complication and that its occurrence and impact differ substantially between patients with and without cancer, as well as among different oncologic subgroups. Overall, AKI developed in about one in five hospitalized patients, but its incidence was significantly higher in those with cancer (30.1% vs. 19.6%). These data confirm that oncologic patients represent a particularly vulnerable population to AKI development [9,17]. Moreover, although the distribution of AKI stages appeared similar across populations, cancer patients more frequently developed stage 3 AKI, suggesting a higher propensity toward severe renal injury once AKI occurs in the oncologic setting. When stratifying by malignancy type, important differences emerged. AKI was most frequent in hematologic cancers, affecting nearly 40% of patients, consistent with earlier reports [18]. This pattern probably reflects the unique pathophysiological context of hematologic cancers, where tumor lysis, high-intensity chemotherapy, sepsis, and more frequent ICU admission may enhance renal risk [19,20]. AKI incidence was lower in non-metastatic and metastatic solid cancers, although still significantly elevated compared with non-oncologic populations. These findings support the view that AKI epidemiology in cancer is not uniform but instead depends strongly on malignancy type and its associated pathophysiology [21]. Across the entire cohort, sepsis and ICU admission emerged as the strongest determinants of AKI development, confirming well-established mechanisms linking hemodynamic instability, systemic inflammation, and renal hypoperfusion to AKI onset in both oncologic and non-oncologic settings [22,23]. Additional factors such as older age, female sex, and heart failure were also associated with increased AKI risk, consistent with prior population studies [24,25]. Interestingly, pre-existing CKD appeared inversely associated with AKI risk. This counterintuitive finding likely reflects the limitations of administrative coding and the exclusion of advanced CKD stages, as well as the possibility that patients with known CKD are monitored more closely and managed with stricter avoidance of nephrotoxins [26]. Regarding diabetes mellitus, no significant association with AKI risk was observed. This result should be interpreted cautiously, as diabetes was identified based on hospital discharge forms, a definition that likely encompasses a heterogeneous population ranging from well-controlled disease to patients with poor glycemic control and multiple comorbidities. In the absence of laboratory data or information on disease severity, the true impact of diabetes on AKI risk may therefore be underestimated.

Notably, each cancer subgroup demonstrated a distinct pattern of AKI risk factors. Hematologic malignancies and non-metastatic solid cancers showed associations with “traditional” predictors, including age, sex, heart failure, and sepsis.

Conversely, among patients with metastatic solid tumors, none of the evaluated variables reached statistical significance in multivariable analysis, despite odds ratios largely consistent with established AKI risk factors. This finding should be interpreted with caution and does not imply the biological irrelevance of conditions such as sepsis or ICU admission. Rather, it likely reflects reduced statistical power (due to the small number of metastatic patients reported with sepsis or admitted to the ICU), wide confidence intervals, and reduced risk discrimination in a population characterized by advanced disease, high baseline vulnerability, and frequent clustering of severe clinical features. In this context, additional acute insults may provide limited incremental prognostic information beyond an already elevated baseline risk.

On the other hand, this observation is consistent with the well-recognized complexity of AKI pathogenesis in advanced cancer, where renal injury results not only from patient-related factors and acute complications, but also from overwhelming systemic disease burden, multiorgan dysfunction, and treatment-related toxicity [27,28].

Together, these findings reinforce the concept that cancer-associated AKI should not be approached as a homogeneous entity. Rather, AKI risk assessment and preventive strategies must incorporate the specific clinical and biological context of each malignancy [29,30]. Outcome analyses further demonstrated the prognostic significance of AKI severity in cancer patients. In the oncologic population, AKI per se did not independently predict in-hospital mortality after adjusting for comorbidities and sepsis. However, AKI severity, particularly stage 3 AKI, remained strongly associated with increased mortality, in agreement with results from large ICU and oncology cohorts [17,31,32].

Interestingly, oncological patients also presented higher sCr levels at discharge, which may represent an important risk for the subsequent development of CKD [33].

Subgroup analysis showed that in hematologic malignancies, only stage 3 AKI independently predicted mortality, whereas in both non-metastatic and metastatic solid cancer, AKI severity remained an important prognostic factor, although attenuated in advanced metastatic disease, where competing mortality risks possibly influence clinical outcomes [34]. These observations have important clinical implications. Hematologic patients require intensified renal monitoring, given their high AKI incidence and frequent exposure to sepsis and nephrotoxic therapies [28,35]. Across all cancer groups, early identification and treatment of infection are central components of AKI prevention, while hydration management, nephrotoxin stewardship, and timely nephrology consultation should be tailored to cancer type and individual risk profile [36]. Moreover, the observation that severe AKI, but not mild AKI, consistently predicts mortality highlights the importance of interventions aimed at early recognizing AKI and preventing its progression [15]. Finally, these findings support a multidisciplinary approach involving oncologists, nephrologists, intensivists, and supportive care specialists, particularly in high-risk populations such as those with hematologic cancers or critical illness [37]. Despite several strengths, including the large real-world sample and specific stratification by cancer type, some limitations should be acknowledged. These include its retrospective design, reliance on administrative coding, and lack of urine output data.

The absence of urine output data is particularly relevant among cancer patients, in whom serum creatinine may be an unreliable diagnostic marker due to malnutrition, sarcopenia, and physical inactivity. This limitation, however, is common to much of the current AKI epidemiological literature [38]. In addition, the category of metastatic solid tumors is intrinsically heterogeneous, as metastatic involvement of different organs may confer markedly different AKI risk profiles. For example, liver or kidney metastases are likely to have a different impact on renal function compared with metastases to other sites [39]. Furthermore, since the original hospital data report system was not specifically designed for this analysis, detailed information on cancer staging, metastatic burden, specific causes of AKI, and oncologic treatment regimens was unavailable. This limitation is particularly relevant given the nephrotoxic potential of several anticancer therapies or procedures, including platinum-based chemotherapy and immune checkpoint inhibitors, and exposure to iodinated contrast media [40]. Future prospective studies should incorporate these variables, especially considering the rapidly evolving oncologic treatment landscape.

## 5. Conclusions

In conclusion, AKI epidemiology and prognosis vary substantially not only between oncologic and non-oncologic patients but also across different malignancy types. Each cancer category exhibits a distinct pattern of AKI risk factors and outcomes. Analyses limited to patient-related factors alone may not fully capture the complexity of AKI in cancer, underscoring the need for future studies that integrate disease burden, treatment-related toxicity, and dynamic clinical parameters.

Recognizing these issues and the peculiarity of different oncological patient groups is essential to guide future research, inform risk stratification, and shape personalized prevention and management strategies for AKI in the oncologic population.

## Figures and Tables

**Table 1:** Characteristics of oncological and non-oncological patients.

	Oncological Patients	Non-Oncological Patients	*p*
**N. of subjects, n (%)**	6723 (11.9)	49,668 (88.1)	
**Age (years)**	73 ± 12.3	69.7 ± 19.4	<0.001
**Sex M**	3780 (56.2)	23,190 (46.7)	
**F**	2943 (43.7)	26,478 (53.3)	<0.001
**Comorbidities, n (%)**			
DM	602 (8.9)	4247 (8.5)	0.254
CKD	422 (6.3)	4088 (8.2)	<0.001
HF	343 (5.5)	4833 (9.7)	<0.001
**sCr admission (mg/dL)**	1.21 ± 1.05	1.13 ± 0.86	<0.001
**Admission ward, n (%)**			
Medical unit	1366 (20.3)	10,937 (22)	
Surgery	743 (11.05)	9362 (18.8)	
ICU	89 (1.3)	2747 (5.5)	
ER	4525 (67.3)	26,622 (53.6)	<0.001
**Sepsis, n (%)**	315 (4.7)	2761 (5.6)	0.003

Abbreviations: DM = Diabetes Mellitus, CKD = Chronic Kidney Disease, HF = Heart Failure, sCr = Serum Creatinine, ICU = Intensive Care Unit, ER = Emergency Room, M = Male, F = Female.

**Table 2:** Characteristics of oncological subgroups.

	Oncological Population			
	Haematological Cancer	Solid Cancer Without mts	Solid Cancer with mts	*p*
**N. of subjects, n (%)**	1107 (16.47)	4767 (70.90)	849 (12.63)	<0.001
**Age (years)**	73.9 ± 11.8	73.1 ± 12.5	71.4 ± 11.7	0.006
**Sex M**	603 (54.47)	2745 (57.58)	433 (51.00)	
**F**	504 (45.53)	2022 (42.42)	416 (49.00)	0.001
**Comorbidities, n (%)**				
DM	86 (7.8)	446 (9.3)	71 (8.3)	0.199
CKD	107 (9.6)	292 (6.1)	23 (2.7)	<0.001
HF	69 (6.3)	318 (6.7)	29 (3.4)	0.001
**SCr admission (mg/dL)**	1.39 ± 1.21	1.21 ± 1.05	1.06 ± 0.71	<0.001
**Admission ward, n (%)**				
Medical unit	297 (26.83)	889 (18.64)	180 (21.22)	
Surgery	52 (4.74)	630 (13.22)	61 (7.15)	
ICU	17 (1.52)	67 (1.40)	6 (0.70)	
ER	741 (66.91)	3181 (66.74)	602 (70.93)	<0.001
**Sepsis**	216 (19.5)	89 (1.86)	14 (1.17)	<0.001

Abbreviations: mts = metastases, DM = Diabetes Mellitus, CKD = Chronic Kidney Disease, HF = Heart Failure, sCr = Serum Creatinine, ICU = Intensive Care Unit, ER = Emergency Room, M = Male, F = Female.

**Table 3:** AKI incidence and staging in oncological vs. non-oncological patients.

	Oncological Patients	Non-Oncological Patients	*p*
**N. of subjects, n (%)**	6723	49,668	
**AKI, n (%)**	2022 (30.1)	9749 (19.6)	<0.001
**Stage 1**	1155 (57.1)	5648 (57.9)	
**Stage 2**	535 (26.4)	2672 (27.4)	
**Stage 3**	332 (16.4)	1429 (14.6)	0.12
**Only AKI stage 3**	-	-	0.04

Abbreviations: AKI = Acute Kidney Injury.

**Table 4:** AKI incidence and staging in oncological subgroups.

	Haematological Cancer	Solid Cancer Without mts	Solid Cancer with mts	*p*
**N. of subjects, n (%)**	1107	4767	849	
**AKI, n (%)**	435 (39.3)	1318 (27.6)	269 (31.7)	<0.001
**Stage 1**	237 (54.6)	769 (58.3)	149 (55.4)	
**Stage 2**	126 (28.9)	331 (25.1)	78 (29)	
**Stage 3**	72 (16.5)	218 (16.5)	42 (15.6)	0.444
**Only AKI stage 3**	-	-	-	0.028

Abbreviations: mts = metastases, AKI = Acute Kidney Injury.

**Table 5:** Multivariate logistic regression for AKI risk development in oncological and non-oncological populations.

	Oncological AKI			Non-Oncological AKI		
	OR	CI	*p*	OR	CI	*p*
**Sex (female)**	1.23	1.04–1.4	0.014	1.21	1.13–1.3	<0.001
**Age**	1.004	0.99–1.01	0.184	1.03	1.02–1.03	<0.001
**CKD**	0.66	0.43–0.99	0.050	0.82	0.72–0.95	0.01
**HF**	1.39	0.9–2	0.076	1.59	1.38–1.75	<0.001
**DM**	0.89	0.6–1.2	0.502	0.87	0.76–1.002	0.05
**Sepsis**	4.96	3.3–7.4	<0.001	5.27	4.6–6.04	<0.001
**ICU**	3.89	2.7–5.6	<0.001	5.96	5.47–6.48	<0.001

Abbreviations: AKI = Acute Kidney Injury, OR = odds ratio; CI = confidence interval; CKD = chronic kidney disease; HF = heart failure; DM = diabetes mellitus; ICU = intensive care unit.

**Table 6:** Clinical outcomes in oncological and non-oncological populations.

	Oncological	Non-Oncological	*p*
**N.**	6723	49,668	
**Mortality, n (%)**	1110 (16.52)	3581 (7.21)	<0.001
**LOS (days)**	12 (7–21)	8 (4–15)	<0.001
**LOS ≥ 15 days, n (%)**	2842 (42.3)	12,541 (25.2)	<0.001
**sCr at discharge (mg/dL)**	1.14 ± 0.90	1.07 ± 0.75	<0.001

Abbreviations: LOS = length of Hospital stay, sCr = Serum Creatinine.

**Table 7:** Multivariate Cox analysis for mortality risk in oncological and non-oncological patients *.

**Mortality Risk in Non-Oncological Patients**						
**Variable**	**Univariate HR**	**CI**	***p***	**Multivariate** **HR**	**CI**	***p***
**Sex (female)**	0.96	0.90–1.02	0.21	0.79	0.74–0.85	<0.001
**Age**	1.02	1.01–1.02	<0.001	1.01	1.01–1.02	<0.001
**AKI**	2.12	1.90–2.35	<0.001	1.52	1.39–1.65	<0.001
**HF**	2.36	2.04–4.79	<0.001	2.10	1.61–2.73	<0.001
**Sepsis**	4.45	3.59–5.42	<0.001	3.68	3.41–3.97	<0.001
**Mortality Risk in Oncological Patients**						
**Variable**	**Univariate HR**	**CI**	***p***	**Multivariate** **HR**	**CI**	***p***
**Sex (female)**	0.92	0.81–1.04	0.14	0.92	0.81–1.03	0.15
**Age**	1.01	1.01–1.02	<0.001	1.01	1.00–1.01	<0.001
**AKI**	1.18	1.03–1.36	0.01	1.06	0.92–1.19	0.49
**HF**	2.02	1.24–4.01	<0.001	1.90	1.57–2.27	<0.001
**Sepsis**	2.64	2.14–3.30	<0.001	2.58	2.17–3.06	<0.001

* Cox analysis performed according to AKI Model 1, i.e., considering the overall AKI development as a risk factor. Abbreviations: HR = Hazard Ratio, CI = Confidence Interval, AKI = Acute Kidney Injury, HF = Heart Failure.

**Table 8:** Outcomes in oncological subgroups.

	Haematological Cancer	Solid Cancer Without mts	Solid Cancer with mts	*p*
**N.**	1107	4767	849	
**Mortality, n (%)**	209 (18.9)	706 (14.89)	195 (22.9)	<0.001
**LOS (days)**	14 (8–24)	12 (7–20)	12 (8–19)	<0.001
**LOS ≥ 15 days, n (%)**	540 (48.7)	1953 (40.9)	349 (41.1)	<0.001
**sCr at discharge (mg/dL)**	1.31 ± 1.08	1.12 ± 0.84	1.06 ± 0.90	<0.001

Abbreviations: mts = metastases, LOS = length of Hospital stay, sCr = Serum Creatinine.

**Table 9:** Multivariate Cox analysis for mortality risk in oncological patients’ subgroups.

Hematological Cancer					
Model	Sex (F vs. M) HR (95% CI)	Age	AKI	Heart Failure	Sepsis
Model 1—AKI overall	0.87 (0.66–1.14)	1.04 (1.02–1.05)	1.03 (0.76–1.39)	1.38 (0.91–2.08)	2.63 (1.97–3.51) §
Model 2—AKI stages	0.67 (0.48–0.95) *	1.03 (1.01–1.05) §	1.20 (0.95–1.51)	1.31 (0.78–2.19)	1.89 (1.33–2.69) §
Model 3—AKI stage 3	0.66 (0.47–0.94) *	1.03 (1.02–1.05) §	1.78 (1.21–2.62) §	1.39 (0.83–2.31)	1.96 (1.38–2.78) §
**Solid cancer without metastases**					
Model					
Model 1—AKI overall	0.90 (0.77–1.04)	1.01 (1.00–1.02) §	1.09 (0.93–1.28)	2.20 (1.76–2.77) §	3.58 (2.76–4.64) §
Model 2—AKI stages	0.82 (0.66–1.01)	1.00 (0.99–1.01)	1.20 (1.05–1.37) *	1.98 (1.41–2.77) §	2.42 (1.78–3.29) §
Model 3—AKI stage 3	0.82 (0.67–1.02)	1.00 (0.99–1.01)	1.22 (0.95–1.57)	1.97 (1.41–2.75) §	2.56 (1.89–3.47) §
**Solid cancer with metastases**					
Model					
Model 1—AKI overall	1.04 (0.78–1.38)	0.99 (0.98–1.01)	0.93 (0.69–1.25)	3.79 (2.03–7.10) §	2.55 (1.28–5.11) *
Model 2—AKI stages	0.78 (0.51–1.19)	1.00 (0.98–1.01)	1.39 (1.07–1.80) *	1.76 (0.53–5.86)	0.99 (0.35–2.79)
Model 3—AKI stage 3	0.79 (0.52–1.20)	1.00 (0.98–1.01)	1.54 (0.94–2.52)	1.93 (0.58–6.40)	1.12 (0.40–3.14)

Abbreviations: HR = Hazard Ratio, CI = Confidence Interval, AKI = Acute Kidney Injury * *p* < 0.05, § *p* ≤ 0.001.

## Data Availability

The datasets used and/or analyzed during the study are available from the corresponding author upon reasonable request.

## Abbreviations

The following abbreviations are used in this manuscript:

AKI	Acute kidney injury
CKD	Chronic kidney disease
CI	Confidence interval
DM	Diabetes mellitus
ER	Emergency room
HDF	Hospital discharge form
HF	Heart failure
HR	Hazard ratio
ICD-9-CM	International Classification of Diseases, Ninth Revision, Clinical Modification
ICU	Intensive care unit
KDIGO	Kidney Disease: Improving Global Outcomes
LOS	Length of hospital stay
MTS	Metastases
OR	Odds ratio
sCr	Serum creatinine
SD	Standard deviation
